# Consumer Motivation to Buy Organic Food Depends on Lifestyle

**DOI:** 10.3390/foods8110581

**Published:** 2019-11-16

**Authors:** Andrzej Soroka, Julia Wojciechowska-Solis

**Affiliations:** 1 Department of Medical Sciences and Health Sciences, Siedlce University of Natural Sciences and Humanities, 08-110 Siedlce, Poland; wachmistrz_soroka@o2.pl; 2 Department of Agrobioengineering, University of Life Sciences in Lublin, 20-950 Lublin, Poland

**Keywords:** organic food consumption, health lifestyle, consumer choice, motives and barriers, discriminant analysis, Polish consumers

## Abstract

The aim of the paper was to determine the relationship between the physical activity undertaken by Polish consumers and their attitude towards organic food. The motives for the selection of organic food, the barriers that consumers face when buying it, and the requirements set by consumers were determined. The research was carried out by means of a diagnostic survey using the author′s questionnaire and the International Physical Activity Questionnaire (IPAQ). In total, 3436 respondents from all over Poland were examined using the selected research sample. Statistica 13.1 PL was used for statistical analyses. The results of the research indicate that the main reasons for the selection of organic food, especially among physically active respondents, were the perceived lack of harmful substances and that it is healthy in itself. The study showed that physically active respondents preferred to purchase, to a greater extent, less processed food produced in a natural way and on organic farms. The main reason for purchasing organic food in physically inactive respondents was due to its taste values. The originality of the paper stems from demonstrating differences and similarities in the motives for buying organic food expressed by physically active consumers and those preferring passive lifestyles.

## 1. Introduction

Consumer behavior has become an important and popular object of scientific research. This is influenced by the growing importance of health care, including concern for a high level of physical fitness. The relationship between health and physical activity and human nutrition has become evident. These are inseparable elements when considering problems related to healthy lifestyle [1,2,3].

Lifestyle consists of certain behaviors as well as the related motives (and the effects as a consequence), goals, or instruments of these behaviors [4,5]. The consumption model is closely related to a healthy lifestyle. It has been shown that consumers are mainly motivated by health considerations and environmental protection in consumption patterns [6].

Conducted research in the field of proper nutrition indicates that there is a constant intensification of lifestyle-related diseases such as diabetes, hypertension, and heart failure, which are directly related to a diet containing high levels of carbohydrates, fats, and refined sugars [7]. This condition is also affected by a lack of physical activity, which is still an underestimated factor affecting health, morbidity, and mortality among people. Lack of physical activity is the fourth leading cause of premature death among the world’s population [8].

Information and promotional/educational activities are mainly aimed at popularizing the right patterns of consumption based on the consumption of food produced in a natural way [9]. Such activities serve to restore the popularity of organic farming, which is also intended to protect the natural environment [10]. The degradation of the natural environment is largely due to an unsustainable pattern of consumption; therefore, a strong emphasis is put on the shift of consumption from conventional products to food produced in organic conditions [11].

Proper nutrition and physical activity have an impact on reducing the risk of civilization diseases the incidence of which has steadily increased in recent decades. According to the International Diabetes Federation, the number of adults suffering from diabetes in the world will increase to 439 million by 2030 [12]. The predictions with respect to overweight and obesity are also worrying [13]. Promoting systematic physical activity is an effective means of promoting health both physically and mentally [14]. A positive correlation between physical activity and mood improvement, better self-perception/higher self-esteem [15], and reduced depression and anxiety states has been proven [16].

The aim of the study was to determine the relationship of physical activity undertaken by Polish consumers and their attitude towards organic food. Goals were set to define:

- RQ1: Motives for the selection of organic instead of conventional foods;
- RQ2: Barriers that consumers face when buying organic food;
- RQ3: Requirements for natural food production.

It was hypothetically assumed that: “There is a statistical relationship between the variables that refer to a healthy lifestyle (as shown by the participation of respondents in physical activity) and the tendency to buy and consume organic products. This applies to purchase preferences, motives, and requirements with respect to organic food”. 

## 2. Material and Methods

The research was carried out by means of a diagnostic survey, using the author′s questionnaire, from which four questions were used in the present study. A five-point Likert scale was used to measure attitudes (where 1 = low importance for consumer and 5 = high importance for consumer), which was preceded by the application of construction and validation procedure. The index of scale reliability was calculated, where Cronbach’s alpha was 0.85. The research was carried out between April and September 2015.

The study conforms to the code of ethics of the World Medical Association and the standards for research recommendations of the Helsinki Declaration. The protocol was approved by the local university ethics committee at the Siedlce University of Natural Sciences and Humanities. To ensure confidentiality, all data were anonymized before analysis. The survey was conducted in the form of telephone inquiries, where the interviewers asked questions to respondents and applied responses to prepared spreadsheets. The standard interview with the respondents lasted 10 min. Two spreadsheets were used, one of which concerned questions related to organic food, while the second one, in the form of an IPAQ questionnaire (International Physical Activity Questionnaire), determined the level of physical activity of the respondent. The IPAQ is particularly recommended for the assessment of the level of physical activity of the entire population [17]. The questionnaire is recognized as one of the most frequently used survey tools for monitoring physical activity [18]. Respondents were classified into one of three levels of physical activity: high, moderate, and low. A low activity level (below 1500 metabolic equivalents of work (MET)-min/week) classified the respondent in the group of inactive persons, while a moderate level of activity indicated a sufficient level, and the group of respondents with a high activity level were considered physically active. Assignment to one of the abovementioned activity levels is made on the basis of energy expenditure expressed in MET-min/week [19]. The MET factor corresponds to the amount of oxygen used at rest and is 3.5 mL O_2_ per kilogram body weight per minute [20]. The total energy expenditure is calculated by multiplying the MET coefficient appropriate for a given activity for its duration expressed in minutes per day and the number of days in a week in which it was take place [19].

A procedure was used in the selection of the sample size where the confidence level was set at 0.95, the estimated fraction size at 0.50, and the maximum error at 0.03. The selection of a representative research sample included: education, sex, and place of residence. Quota sampling was applied in which the respondents were selected on the basis of availability until the number of completed surveys was exhausted.

In total, 3436 respondents from all over Poland were examined. Three groups of respondents were created considering their education. In each of the three groups of respondents, a representative research sample was selected of 1066 individuals for each group. Due to the possibility of rejecting some of the surveys in each group, the number of respondents was increased by about 50 to 100. A representative sample was selected in each of the three groups of respondents. This allowed the selection of 1150 respondents with primary and vocational education, 1180 with a secondary education, and 1106 with higher education. Random stratification was used in each of the groups during the sample formation procedure. The population was divided according to the place of residence: villages, cities with up to 30,000 residents, cities with over 30,000 residents, and by gender. The number of respondents in each of the surveyed groups taking into account their place of residence and gender was determined in proportion to the number of adult residents of Poland, which excludes the possibility of other moderating variables [21].

Statistica 13.1 PL (StatSoft Inc., Tulsa, OK, USA) was used for statistical analyses and the implemented discriminatory function, which was used to resolve which variables discriminated two emerging groups, with respondents who declared physical activity and who reported a lack thereof. The classification function was applied in the form of coefficients that were defined for each group. Prior to the analysis, multivariate normality was analyzed, testing each variable for normal distribution. It was assumed that variable variance matrices are homogeneous in groups. Slight deviations were not that significant due to the large number of respondents in particular groups. Means for which the probability was less than *p* < 0.05 were considered statistically significantly different.

## 3. Results

The desire to consume healthy food was the main motive for organic food selection among respondents. The respondents who reported participation in physical activity, in whom the value of the classification function was 1.297, paid attention to such food to a significantly higher degree, at *p* < 0.001, while among physically inactive respondents this value was 1.049. The respondents commented on the content of harmful substances in organic food in a similar manner. At *p* = 0.010, higher values of the classification function were found in physically active respondents, who also paid significantly more (*p* < 0.001) attention to care for the natural environment than respondents who declared lack of physical activity. The motive concerning a concern for healthy nutrition of one’s family was at a similar level in both groups of respondents in the created discriminant function model. The respondents declaring a lack of physical activity emphasized one motive significantly more (*p* < 0.001), more strongly indicating the purchase of organic food due to the search for childhood flavors, i.e., sensory attributes, than did physically active respondents (Table 1).

Eggs were the most commonly purchased organic products, which significantly more frequently (*p* = 0.025) were bought by physically active respondents than those who declared a lack of activity. Physically active respondents also reported, to a significantly higher degree (*p* = 0.006), purchasing fresh vegetables and their preserves. Pork and its products from organic production were purchased at a similar level, with high values of the classification function in both groups. Those who declared physical activity significantly more often purchased cereal products (*p* = 0.004) and goat’s milk and its products (*p* = 0.002). The respondents declaring a lack of physical activity bought more of these types of products only in case of cow’s milk and derived products (Table 2).

Considering the barriers faced by respondents when buying organic food, too high prices were indicated most frequently in both groups of respondents. A general difficulty with the purchase of such food in the context of its availability was another problem, particularly with respect to physically active respondents (*p* = 0.019) as opposed to the physically inactive. Inactive respondents indicated the lack of trust in organic food, lack of skills in distinguishing organic food from conventional food, and no attachment to such purchases, i.e., indifference to organic products in a significantly higher degree, at *p* < 0.001 (Table 3).

Higher needs regarding the requirements for organic food by both groups of subjects were expressed by respondents describing themselves as physically active people. A significant difference in the size of the classification function occurred at *p* < 0.001 in both cases. This group of respondents also paid more attention (*p* < 0.001) to the production method, i.e., low levels of processing, and thus to a shorter shelf-life of organic products and their low salt content. The packaging of purchased products, which should be environmentally friendly, i.e., self-degradable, was very important for this group of respondents. The group of respondents a declaring physically inactive lifestyle acknowledged to a greater extent taste attributes in organic products. Both groups paid similar attention to the low amount of fat in organic food and the opinion that organic products are richer in nutrients compared to conventional products (Table 4).

## 4. Discussion

It is well known that the consumption of food has a significant relationship with the environment, individuals, and public health [22]. Consuming food with undesirable residues and microorganisms causes severe individual health problems [23]. Food-borne diseases result in medical costs and losses to the public health sector [24]. Hence, promoting and accelerating the adoption of more sustainable food behaviors is of the utmost importance for enhancing environmental sustainability as well as individual and public well-being. Sustainable food behaviors include activities such as purchasing and consuming organic food, eating less unhealthy food, eating local food, and preparing food that has less wastage [25]. The growth income has driven consumer demand for food products, and this is especially true in developing countries, for example Poland, particularly for healthy and environmentally friendly food [26].

Organic food purchase and consumption has been widely regarded as contributing to sustainable behavior [27]. This is partly driven by consumers′ socio-environmental responsibility in addition to their personal interest and choice. The majority of consumers believe that organic food is eco-friendly, healthier, cleaner, more nutritious, tastier, and safer as compared to conventional food [28,29].

Demand for organic food products is growing worldwide. Health issues are often the main motivation for consumers to buy them [24]. The research hypothesis assumed that respondents participating in physical activity were more likely to buy organic food than physically inactive respondents. The hypothesis was confirmed, and physically active respondents were concerned with the protection of the natural environment. Problems related to environmental protection are currently being raised and implemented not only by ecologists but also by the dynamically developing food industry. In this case, not only economic costs are considered but also the opinions of consumers who demand the protection of natural assets. It is they who, through their opinions that affect the sale of a given product, put pressure on producers [30]. These are the consumer choices that nowadays affect the strategies of food-producing enterprises [31,32,33], as well as policies aimed at health and environmental protection [34].

The research confirmed the greater likelihood of physically active people to purchase organic food, consistent with our research hypothesis regarding the assessment of organic food as more healthy than conventional foods and not containing harmful substances. This group of respondents also defined themselves caring more for the natural environment by supporting the production of food in organic conditions.

The desire to support local organic farms, which largely sell their production directly to a local customer, was an additional important problem often declared by consumers next to health criteria and care for the natural environment, which consumers considered during shopping [35]. Such actions have a positive impact on the protection of local natural resources [36,37].

The motives for the preference of organic food were also reflected in the type of food consumed. In almost all cases, the higher declarations of purchasing organic food were indicated by physically active respondents. It seems that healthy food combined with physical activity is the basic condition for maintaining a healthy lifestyle and taking care of their health for this group of respondents. Such results were also a confirmation of the research hypothesis.

The education of respondents [38,39] is important when choosing organic products. It is suggested that the purchase of food such as meat and potatoes, i.e., basic products, is made more by less educated people, while fresh vegetables and their preserves, honey, and eggs (more frequently in their ecological form) are preferred purchases of people with higher education who also have a higher degree of need to participate in physical activity [40]. According to this group of consumers, organic food contains more vitamins and minerals, and at the same time fewer undesirable substances than traditional products [41].

Barriers associated with the purchase of organic food definitely include too high prices for such products. This problem was raised to a large extent in both groups of respondents. It was pointed out that physically inactive respondents did not pay much attention to the purchase of organic food, which can be evidenced by declaring the lack of trust in such food and not attaching importance to its purchase. Research carried out by Rödiger and Hamm [42] showed that 17% of the population was responsible for 76% of all organic food purchases, which may indicate that a small percentage of the public is interested in purchasing organic products. This group of respondents also had a greater problem with distinguishing organic food from conventional food. It is indicated that there is not enough organic food on the market because consumers have a problem with distinguishing it from the generally sold product range [43]. It is indicated, as confirmed in the conducted research, that there are differences between groups of consumers (in this study, the attitude to physical activity, where definite higher requirements for organic food were reported by consumers declaring higher physical activity) as to the frequency of organic product purchases [44]. According to Qasim et al. [45] consumption values and self-identity are the essential antecedents of sustainable consumer behavior. By integrating the theory of consumption values and the self-identity approach, their research showed the relationship between consumption values, environmental self-identity, and the behavioral intention to consume organic food.

The importance of organic food was confirmed by the introduction of the concept of sustainable development, which was created as a response to the rapid growth in the popularity of organic food products [46,47,48,49]. The sustainable nutrition regime has also been defined by the Food Agriculture Organization as nutrition oriented towards the protection of biodiversity and ecosystems. The concept refers to foods that are culturally acceptable, available, properly nutritious, and safe for health, with natural and human resources optimized for their production [50].

Research confirmed that physical activity concerns people with high needs of proper functioning in modern society, where a strong emphasis is placed on a healthy lifestyle based on the consumption of organic food and life in conditions close to organic.

## 5. Conclusions

The main motives for the selection of organic food are a perception of a lack of harmful substances in such products and the belief that such food is healthy in itself. Greater attention to the consumption of organic products was indicated by physically active respondents, who focused on the health motive to a greater extent. The taste of organic products, including the search for flavors of childhood, was an important motive for respondents declaring lack of physical activity.

The main problem with the purchase of organic food concerns its price, which in the general opinion is too high. Respondents declaring their activity were less concerned with problems associated with a lack of trust in organic food, a lack of inability to distinguish organic products from conventional products, or not attaching much importance to such food.

Subjects participating in physical activity paid more attention to the fact that organic food was less processed and produced in a natural manner on organic farms. The main reason for purchasing organic food in physically inactive respondents was its taste qualities.

## Figures and Tables

**Table 1:** Motives for the selection of organic food by Polish residents, including declared physical activity.

Motives Associated with Organic Food Selection	Model of Discriminant Analysis			Classification Function	
	Wilks′ Lambda: 0.563			Declared Physical Activity	
	Wilks′ Lambda	F Value	*p* Value	Active	Inactive
Searching for childhood flavors	0.563	15.389	0.001 *	0.844	1.135
Such food is healthy	0.602	9.538	0.001 *	1.297	1.049
Does not contain harmful substances	0.598	6.621	0.010 *	1.199	1.065
I care about healthy nutrition of my relatives	0.576	2.207	0.138	0.980	0.894
I care about the natural environment	0.582	10.291	0.001 *	0.987	0.662
Constants				12.309	11.562

* Level of significance at *p* < 0.050. Source: Authors′ own analysis based on study material.

**Table 2:** Type of purchased organic food.

Type of food	Model of Discriminant Analysis			Classification Function	
	Wilks′ Lambda: 0.623			Declared Physical Activity	
	Wilks′ Lambda	F Value	*p* Value	Active	Inactive
Fresh vegetables and their preserves	0.652	7.553	0,006 *	1.641	1.476
Cereal products	0.638	8.310	0.004 *	1.124	0.970
Goat’s milk and its products	0.687	9.250	0.002 *	1.236	1.085
Eggs	0.659	4.990	0.025 *	1.722	1.600
Cow’s milk and its products	0.632	5.329	0.021 *	1.059	1.184
Pork and its products	0.629	1.270	0.259	1.541	1.502
Constants				15.987	14.802

* Level of significant difference at *p* < 0.050. Source: Authors′ own analysis based on study material.

**Table 3:** Barriers affecting the purchase of organic food.

Type of Barriers Related to the Purchase	Model of Discriminant Analysis			Classification Function	
	Wilks′ Lambda: 0.583			Declared Physical Activity	
	Wilks′ Lambda	F Value	*p* Value	Active	Inactive
I do not trust organic food	0.582	17.025	0.001 *	0.577	0.990
There are many problems with the purchase of such food	0.562	5.465	0.019 *	1.312	1.147
I cannot distinguish organic food	0.591	11.566	0.001 *	0.567	0.921
Organic food is too expensive	0.604	2.282	0.131	2.328	2.421
I do not pay much attention to the purchase of such food	0.589	16.289	0.001 *	0.515	0.944
Constants				8.731	10.077

* Level of significant difference at *p* < 0.050. Source: Authors′ own analysis based on study material.

**Table 4:** Requirements for organic food set by respondents.

Type of Requirements	Model of Discriminant Analysis			Classification Function	
	Wilks′ Lambda: 0.654			Declared Physical Activity	
	Wilks′ Lambda	F Value	*p* Value	Active	Inactive
Low-processed, short shelf-life	0.621	28.718	0.001 *	1.203	0.856
It should contain low salt content	0.658	16.777	0.001 *	0.456	0.158
Produced in a natural way	0.679	20.672	0.001 *	1.472	1.071
Production on an organic farm	0.628	21.452	0.001 *	1.468	0.998
Environmentally friendly packaging	0.687	12.569	0.001 *	0.905	0.508
Rich in nutrients	0.659	3.827	0.058	0.846	0.714
High taste values	0.634	4.911	0.038 *	1.140	1.356
It should contain low fat content	0.639	1.487	0.226	0.740	0.644
Constants				16.850	15.377

* Level of significant difference at *p* < 0.050. Source: Authors′ own analysis based on study material.
